# Histopathologic and ancillary findings of subcentimeter thyroid nodules diagnosed as follicular neoplasms: a retrospective institutional study

**DOI:** 10.1007/s00428-025-04308-x

**Published:** 2025-10-20

**Authors:** Angela Feraco, Belen Padial Urtueta, Qianqian Zhang, Luisa Cioni, Alfredo Pontecorvi, Marco Raffaelli, Guido Fadda, Antonino Mulè, Liron Pantanowitz, Esther Diana Rossi

**Affiliations:** 1 Division of Anatomic Pathology and Histology-Fondazione, Policlinico Universitario “Agostino Gemelli” - IRCCS, Università Cattolica del Sacro Cuore, Largo Francesco Vito, Rome, 1 - 00168 Italy; 2 Division of Endocrinology-Fondazione, Policlinico Universitario “Agostino Gemelli” - IRCCS, Rome, Italy; 3 Division of Endocrine Surgery-Fondazione, Policlinico Universitario “Agostino Gemelli” - IRCCS, Rome, Italy; 4 Anatomic Pathology and Histology - Department of Human Pathology in Adult and Developmental Age “Gaetano Barresi”, University of Messina, Messina, 98125 Italy; 5 Department of Pathology, Pittsburg University, Pittsburg, PA USA

**Keywords:** Fine needle aspiration cytology, Indeterminate lesions, Thyroid malignancies, Immunocytochemistry, Personalized medicine, Follicular neoplasm

## Abstract

Thyroid nodules are frequently encountered, mostly detected during ultrasound evaluation of the head and neck, frequently performed for other reasons. Albeit most nodules arise in the setting of multinodular goiter, hypoechoic subcentimeter nodules can be recognized that show suspicious features, requiring them to undergo fine needle aspiration cytology (FNAC) evaluation. ATA guidelines suggest performing FNAC only on nodules greater than 1.5 cm. Nevertheless, the issue emerges if these subcentimeter lesions are aspirated and diagnosed as follicular neoplasms (FN). Herein, we describe an algorithm used to approach such subcentimeter thyroid nodules in our tertiary medical center. All subcentimeter thyroid nodules sampled by FNAC were retrieved, performed between 2014 and 2023 with a diagnosis of indeterminate lesion of high-risk malignancy (Italian Classification system) that corresponds to follicular neoplasm (FN) in the Bethesda Reporting System. These cases were processed only with liquid-based cytology (LBC) with immunocytochemistry (ICC) and molecular testing performed when necessary. The series included 174 indeterminate subcentimeter nodules analyzed with FNAC, based on suspicious ultrasound criteria. The cytological diagnosis included 101 cases with atypia of undetermined significance (AUS) and 74 FN. All FN cases underwent surgery, and the subsequent histological diagnoses revealed 24 (32%) benign and 49 malignant lesions including 38 papillary thyroid carcinoma (PTC) and its variants as well as 11 cases of invasive follicular variant of PTC (I-FVPTC). Furthermore, 30.6% (15/49) of these malignant lesions had lymph node involvement, and 34.6% were multifocal. Among the histological malignant cases, only three (6.1%) cases had a moderate positivity for VE1-BRAF, with eight (16.3%) cases showing a concordant positive HMBE1 and Galectin-3 panel. Although some guidelines do not recommend sampling subcentimeter thyroid nodules, in clinical practice, these may undergo FNAC to help elucidate concerning ultrasound findings. In our series, in the presence of suspicious ultrasound criteria, 66% of the nodules turned out to be malignant. Although ICC is unable to help make a definitive diagnosis, it serves as a useful pathology ancillary tool in the algorithmic work-up of subcentimeter thyroid lesions.

## Introduction

The detection of nodules in the thyroid gland is a common finding, especially with ultrasonographic (US) examination [1–5]. The ease of performing a thyroid US has consequently led to a 15-fold increase in thyroid cancer diagnosis. The prevalence of thyroid nodules ranges between 3 and 80%, depending on the screening programme and patient population evaluated. Thyroid cancer occurs in a small proportion of patients with thyroid nodules, ranging from 5 to 20%, with a higher incidence in females than males [5–7]. In particular, US evaluation of the thyroid has increased the detection of small subcentimeter nodules with suspicious or clearly malignant imaging features (8–10). However, to avoid an overdiagnosis of thyroid cancer, more strict guidelines are necessary for the management of these subcentimeter nodules [8–10].

According to various guidelines [11–14] it is well known that in the majority of cases, a conclusive diagnosis of the nature of thyroid lesions is likely to be defined by fine needle aspiration cytology (FNAC) together with US [15–23]. Published data have shown that thyroid FNA is able to render a conclusive diagnosis in greater than 80% of cases, thereby guiding the correct clinical and/or surgical management for patients presenting with a thyroid nodule [5–7]. However, it is also important to underline that 20% of the thyroid lesions belong to the categories of indeterminate proliferations, for which, regardless of the nodular size, morphology alone and/or with ancillary techniques is not able to make a conclusive diagnosis [15–23].

Nevertheless, the advantages of FNAC include easy access, a rapid and safe procedure, with very few complications. The majority of thyroid classification systems, as well as the American Thyroid Association (ATA) guideline, have paid attention mainly to thyroid nodules that are larger than 1.5 cm [3, 6, 8–17]. However, even smaller thyroid nodules can be concerning. Indeed, there have been a growing number of subcentimeter nodules that, when surgically removed, yielded a malignant histological diagnosis [16–22]. Despite the fact that some guidelines, including those from the ATA [6], do not suggest performing a FNAC on small nodules, in clinical practice, there is an increasing number of FNACs being performed on subcentimeter thyroid nodules based on suspicious US findings. The most worrisome US findings leading to a FNAC are represented by microcalcifications, spiculated or irregular margins, a taller-than-wide shape, extracapsular extension, and chaotic or increased intranodular vascularity. Hence, a solid hypoechoic nodule also increases suspicion for malignancy. The challenge with such cytological specimens is to appropriately classify these subcentimeter nodules as follicular neoplasms (FN) or at least suspicious for malignancy (SFM). According to the guidelines, the diagnosis of a FN leads to a lobectomy as the gold standard of management. While a diagnosis of benign lesion or overtly malignant subcentimeter lesions includes follow-up and surgery respectively, on the other hand, a subcentimeter lesion diagnosed as FN raises some concerns about the most appropriate management.

Herein, we aim to analyze our FNAC series of subcentimeter-sampled thyroid nodules, diagnosed as follicular lesions. This article also shares our cytologic algorithm approach in these cases to provide data for future guidelines.

## Material and methods

A retrospective, computerized search of all indeterminate subcentimeter thyroid lesions that had undergone FNAC with subsequent histological follow-up was conducted, analyzing the files of the Division of Anatomic Pathology and Histology of the Catholic University, “Agostino Gemelli” Hospital of Rome (Italy) between January 2014 and February 2023. All benign and malignant subcentimeter lesions were excluded from the purpose of our paper. In our institution, all subcentimeter thyroid nodules were evaluated under sonographic guidance (US). Following FNAC, all cases were processed with the liquid-based cytology (LBC) ThinPrep 2000™ method (Hologic Co., Marlborough, MA). To note, since 2000, all thyroid FNACs are processed with only LBC as described in several papers of ours [24, 25]. Each FNAC was performed with two passes devoted to each lesion, using 25 to 27 G needles without any rapid adequacy assessment of the procured material.

The nodules ranged in size from 0.3 to 1.0 cm, and they were all discovered during routine US thyroid check-up performed in the “Centre for Thyroid Diseases” of the Departments of Endocrinology and Endocrine Surgery of the Catholic University. All the patients had been appropriately informed about the procedure and processing of their aspiration samples and written informed consent was signed by each patient. Our study followed the tenets of the Declaration of Helsinki, and we received internal ethics approval.

The LBC protocol utilized has been described in detail in previous publications [24, 25]. For the preparation of any additional slides and further ancillary techniques, such as immunocytochemistry (ICC) and molecular testing, any remaining material was stored in PreservCyt™ solution at room temperature for 3–4 months. Ancillary techniques can be performed when residual cytology material is at 2 ml eluted in 5 ml of PreservCyt solution. Specimen adequacy was defined according to the Bethesda system for reporting cytopathology (TBSRTC) and the Italian classification system, by the presence of at least six groups of epithelial cells within the submitted slides, each of them with at least 10 well-visualized epithelial cells [11, 14].

The final cytologic diagnoses were signed out according to the Italian Working Group SIAPEC-IAP classification system and then reclassified for scientific purposes according to TBSRTC [11, 12, 14]. Albeit that there are few differences among these classification systems, this is not the case for the follicular neoplasm (FN) category [11, 12, 14] showing the same morphological criteria (follicular/microfollicular clusters of medium size thyrocytes with hyperchromatic round nuclei and moderate cytoplasm) in both classification systems. At our institution, all cytological and histological slides with equivocal or doubtful clusters of follicular cells were submitted for consensus review by more than two cytopathologists until a final agreement was achieved. The follow-up for enrolled cases in this study included a period between 12 and 120 months (median 58 months). Specifically, due to the interval between 2013 and February 2023, we adopted the second edition of TBSRTC, so that the atypia of undetermined significance cases (US) are not subclassified as for the more recent 3rd edition into AUS-Nuclear atypia and AUS-Other.

Furthermore, the distribution of all subcentimeter nodules in the same interval (2013–February 2023) included 29 non-diagnostic, 32 benign, and 102 malignant lesions. The morphological features and diagnostic criteria were identical to those larger than 1 cm.

## Immunocytochemistry

ICC staining of slides was carried out with the avidin–biotin peroxidase complex using a selection of specific antibodies (in details HBME-1, Galectin-3, BRAF VE1). The immunocytochemical method, as well as the markers, was standardized according to the manufacturer’s protocol adopted in our institution and previously published [24, 25]. For BRAF VE1 stains, molecular confirmation was performed on the same LBC stored material [26–28].

## Histology follow-up

All surgical pathology specimens were fixed in 10% buffered formaldehyde, embedded in paraffin, sectioned into 5-µm-thick slides, and then stained with hematoxylin–eosin (H&E). Thyroid lesions were classified according to the 2022 WHO Classification of Tumors of Endocrine Organs [29–31]. For the definition of benign lesions, we adopted the term “thyroid follicular nodular disease”. For the definition of tall cell variant/subtype (TCV) of PTC, we included cases of PTC with equal to or more than 30% TCV component with follicular cells that are at least 3 times taller than they are wide. The histological diagnosis of non-invasive follicular thyroid neoplasm with papillary-like nuclear features (NIFTP) was rendered according to the criteria described by Nikiforov et al. [32]. All malignant cases were staged according to the eighth edition of the tumor-node-metastasis (TNM)-based staging system recommended by the American Joint Commission on Cancer (AJCC) [33]. These histological cases were also classified according to the ATA risk categories [6], as described below in the result section.

## Results

This study included only indeterminate subcentimeter thyroid lesions diagnosed as an FN according to TBSRTC and TIR3B according to the Italian classification system [11, 12, 14]. As already mentioned, the two classification systems share the same morphological criteria for the diagnosis of FN and TIR3B. During the same time period, we diagnosed 101 subcentimeter nodules as TIR 3 A (Italian classification system) or AUS (TBSRTC). Only 10 out of 101 underwent surgery due to aesthetic reasons and size; the remaining 91 cases were not submitted to surgery and followed up with repeated FNAC according to the ATA guidelines [6]. Specifically, 56 of these TIR3A/AUS cases had a second FNAC with a new cytologic diagnosis changed to a benign nodule. None of these repeated TIR3A/AUS cases had a diagnosis changed to suspicious for malignancy and/or malignant. The 10 TIR3A/AUS with surgical follow-up resulted in 7 thyroid follicular nodular diseases and 3 follicular adenomas (FA), confirming the benign cellular and architectural criteria used for this category.

However, our study-series included and is focused on 74 cytology samples (3.8% of total FNAC) diagnosed as FN. The patient demographics and clinical-pathologic features for these cases are described in Table 1. We included all cytological samples diagnosed as FN with histological follow-up. The FN series included 19 male and 55 female patients with a median age of 49 years (range 16–83 years and mean: 49.9 years). In this subset of patients, their thyroid neoplasms ranged in size from 4 to 10 mm. Specifically, in eight cases, the FN size ranged between 3 and 5 mm, and in 66 cases between 6 and 10 mm (Table 1). All subcentimeter lesions were discovered incidentally during US radiologic screening for causes unrelated to the thyroid gland and/or thyroid screening evaluation. These nodules were also scored according to any radiologic suspicious criteria. FNAC was performed regardless of the nodule size and based on suspicious radiological criteria including solid and hypoechoic nodules, evidence of microcalcifications, spiculated or irregular margins, a taller-than-wide shape, extracapsular extension, and chaotic or increased intranodular vascularity. The clinical-pathological data did not show any peculiar statistical correlation between size and the different analyzed parameters.

**Table 1:** Summary of clinical-pathological data

Clinical-pathological features	Proportion (*n* = 74 cases)
Age	
Mean	49.2 years
Median	49 years
Range	5–85 years
Gender	
Male	19 (25.6%)
Female	55 (74.4%)
Size	
0–5** mm**	8 (10.9%)
6–10** mm**	66 (89.1%)
Cytology diagnosis *	
FN/TIR 3B	74
Histopathology diagnosis	
Benign^*^	24 (32.4%)
Malignant	49 (66.2%)
NIFTP	1 (1.3%)

*FN* follicular neoplasm according to the Bethesda categories; TIR3B according to the Italian system

*101 AUS (atypia of undetermined significance) were included as negative control; *NIFTP* non-invasive follicular thyroid neoplasms with papillary-like nuclear features

**Fig. 1:**
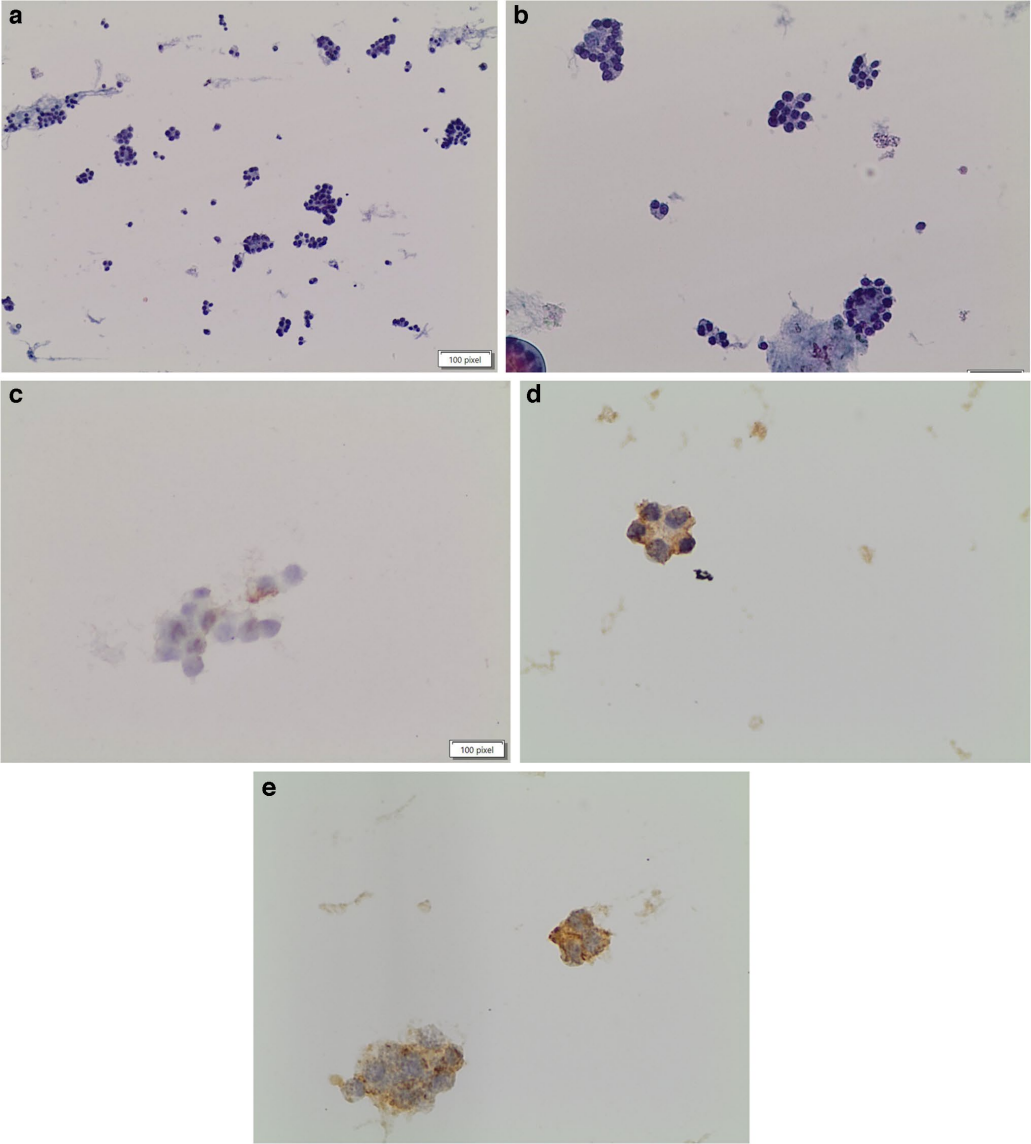
**a**,** b** Follicular neoplasm mostly composed of clusters of follicular cells, medium-sized groups, with a microfollicular architecture (liquid-based cytology, Pap stain × 20 and × 40). **c** Positivity for HBME-1 in one of the clusters; **d** positivity for Galectin-3; **e** Positivity for VE1-BRAF (**a** and **b** × 40)

**Fig. 2:**
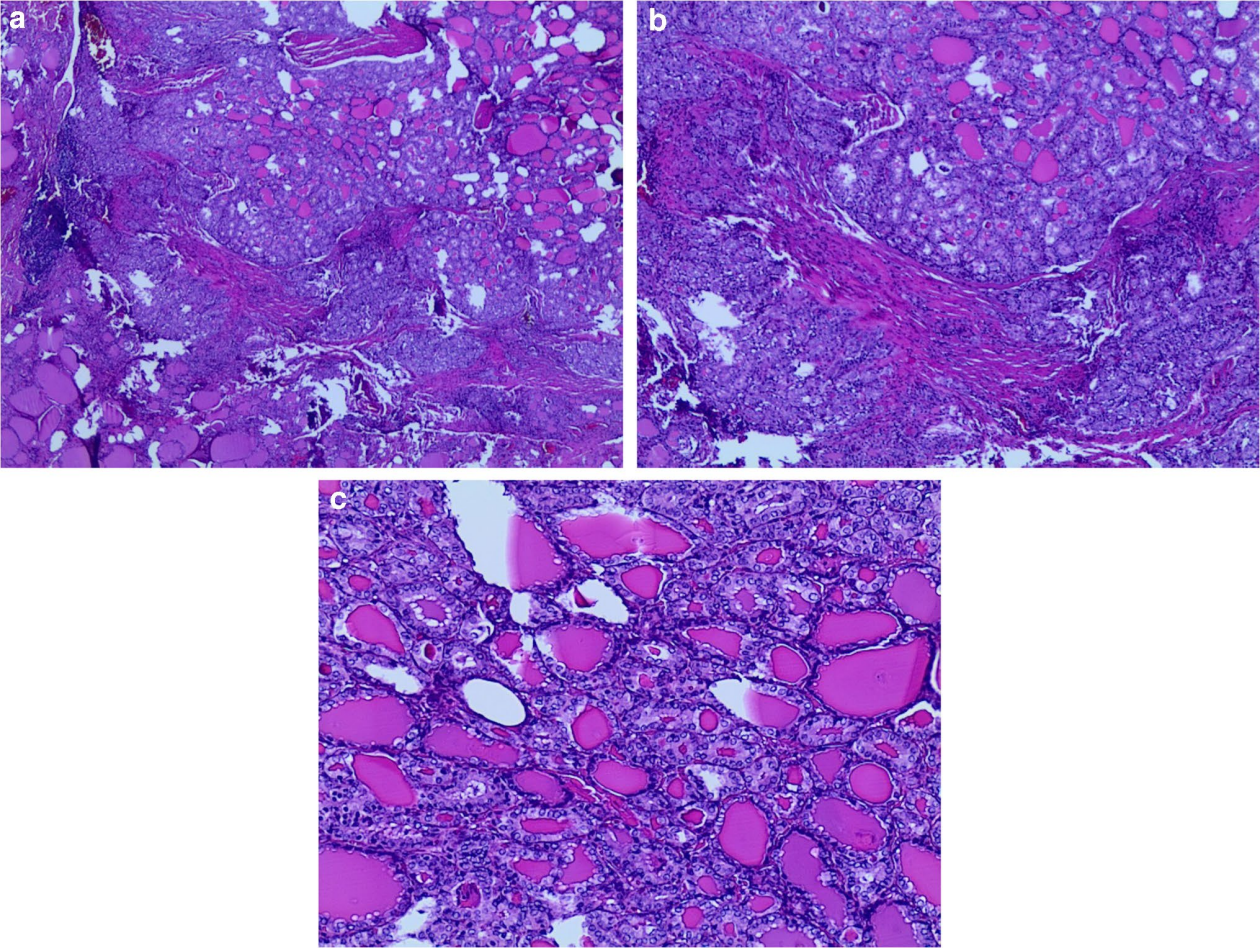
**a**, **b** Histological details of a follicular variant of papillary thyroid carcinoma encapsulated with minimal capsular invasion (**a**); while **b** shows the nuclear details of the lesion (H&E × 20 and × 40)

The histological follow-up of the 74 FN (Fig. 1a–e) cases included benign histology in 24 cases (32.4%), a malignant diagnosis in 49 cases (66.2%), and one NIFTP case (1.3%) (Table 2). The 24 benign cases included 14 diagnosed as “thyroid follicular nodular disease” and 10 subcentimeter follicular adenomas. The 49 malignant cases included only PTC and its subtypes, distributed as follows: 38 classic PTC, two cases of PTC with 15% tall cell features, two PTC cases with 20% solid component, and one PTC with 5% hobnail subtype component. In addition, this subgroup also showed 11 cases with I-FVPTC, including two infiltrative FVPTC and nine minimally invasive encapsulated FVPTC (Fig. 2a, 2). The histological evaluation of these 49 cases showed that 17 (34.6%) had multifocal PTC (15 classic PTC and 2 I-FVPTC), and 15 (30.6%) had lymph node metastases involving a range of lymph nodes between 1 and 4 (9 classic PTC, 5 PTC with more aggressive features, 1 I-FVPTC). To note, the lymph nodes were removed during surgery based on the clinical intraoperative evidence of thicker and larger size. All the 49 malignant cases belonged to the low ATA score risk. To the best of our knowledge, follow-up, based on clinical and radiological evaluations with US, has shown no recurrences or metastases.

**Table 2:** Cyto-histological correlation (74 FN cases) according to TBSRTC

	Goiter	FA	NIFTP	PTC	-FVPTC
FN (74 cases	14	10	1	11°	38

*FN* Follicular Neoplasm, *FA* Follicular adenoma, *PTC* Papillary thyroid carcinoma

°including PTC subtypes 6 classic PTC, two cases of them showing a 15% tall cell subtype component, 2 PTCs with a solid component of 20%, and one PTC with 5% hobnail subtype PTC component. I-FVPTC Infiltrative follicular variant of PTC including 9 invasive FVPTC and 29 minimally invasive encapsulated FVPTC

Table 3 presents the expression results of ICC with a panel of stains made up of Galectin-3, HBME-1, and BRAF VE1 in our series of 74 FN cases (Table 3). By comparison, the subset of cases within the AUS category showed 100% negativity for BRAF VE1, while five cases had weak positivity for HBME-1 and three cases weak positivity for Galectin-3. None of the AUS cases had a concordant positivity for the HBME-1 and Galectin-3 immunomarkers. The 74 FN cases showed that none of the histologically benign lesions (24 out of 74 cases) and the NIFTP case had ICC expression for any of the biomarkers. For the 49 FN cases that turned out to be histologically malignant, we found that 11 cases stained positive for HBME-1 (Fig. 1c) and 13 were positive for Galectin-3 (Fig. 1d), with only eight cases showing a combined positive immunopanel. The evaluation of the VE1-BRAF immunostain was negative for all 24 of the histologically benign cases, as well as for the NIFTP case. In the group of FN cases that were histologically malignant, we found only three (6.1%) cases with moderate VE1-BRAF expression in some of the cells (Fig. 1e). Based on the BRAF-VE1 positivity only in some clusters (less than 10% of the global neoplastic cells), the diagnosis of FN was not changed. However, in the sample report, we reported the focal expression for VE1-BRAF. Furthermore, these three cases underwent total thyroidectomy instead of lobectomy as for all the other wild-type cases. At the histological examination, thesethree VE1-BRAF positive cases had tall cell and hobnail components, confirming, even in this small series, that immunoreactivity is more commonly seen in more aggressive PTC histotypes.

**Table 3:** Immunocytochemical expression in our 74 cases

Diagnosis	VE1-BRAF	HBME-1	Galectin-3	HBME-1 plus Galectin-3
FN (74 cases)	3^§^	11*	13*	8*
	Mild/Moderat***e*** = 3			

*FN* follicular neoplasm

^§^moderate expression of VE1-BRAF

^*^expression only in the group of follicular neoplasms-FN with a malignant diagnosis

To note, the molecular evaluation for *BRAFV600E* mutation was performed on the histological cases with moderate positivity, confirming the expression and in agreement with the ICC results. We did not perform any further molecular evaluation.

## Algorithm approach

As illustrated in the aforementioned result, at our institution, we accordingly follow this algorithm:

- We evaluate all thyroid nodules, including subcentimeter lesions, with US analysis.
- If these lesions, regardless of their size, show suspicious criteria on US, then we perform a FNAC.
- Cytomorphological diagnoses of FN and AUS are supported by additional ancillary studies including ICC and molecular testing, mostly for *BRAFV600E* in order to determine if lobectomy is needed in cases of FN.

## Discussion

Thyroid nodules are increasingly encountered and their prevalence ranges between 3 and 80% according to screening programs and patient population [1–5, 34]. The detection of thyroid nodules is high in endemic goiter areas. Based on US analysis, even in non-endemic areas, the incidence ranges from 20 to 70% [7, 14, 17, 18, 22]. In these last decades, we have witnessed an increasing number of incidental thyroid nodules, including small subcentimeter lesions detected on routine screening during US evaluation. Around 90% of all thyroid nodules are in fact non-palpable. Moreover, neither the number of thyroid nodules nor their size appears to be predictive criteria for malignancy [1–5, 8–10]. The decision whether to perform a FNAC is usually dictated by clinical guidelines, mostly based on nodule size. For instance, the ATA recommends that FNAC be carried out for thyroid nodules measuring > 1.5 cm with low-suspicion sonographic patterns or > 1.0 cm with high/intermediate-suspicion features on US [6]. Routine biopsy of nodules < 1 cm is typically not recommended [6, 35]. Nevertheless, as already mentioned, subcentimeter nodules often still undergo FNAC in clinical practice, as is the case at our institution when US findings in these nodules are concerning. Guidelines for subcentimeter nodules typically advocate that they undergo active surveillance rather than an immediate FNAC or surgical procedure, which does not appear to affect disease-specific survival. However, further work-up is acceptable for subcentimeter nodules if there are specific conditions such as suspicious parameters on US, lymph node and/or distant metastases, or extrathyroidal extension, which all indicate more aggressive disease [6, 35]. The US detection of very small thyroid nodules has increased the incidence of thyroid cancers, even though malignant subcentimeter nodules have low disease-specific mortality [7–10]. According to the ATA and some Asian guidelines, active surveillance instead of surgery is even recommended as a reasonable option for papillary microcarcinoma [6].

Our data showed that 42.3% (74/175) of the indeterminate subcentimeter lesions in our series were diagnosed as follicular neoplasms. The majority of the subcentimeter indeterminate lesions belonged to the AUS group, which have been followed up with active surveillance based on the repetition of FNACs and, when needed, the support of ancillary techniques such as ICC and/or molecular testing. None of our AUS cases had surgery or resulted in a later malignant cytological diagnosis. On the other hand, all 74 of the patients with a subcentimeter FN underwent surgery. Most of these patients had a lobectomy, followed by intra-operative evaluation of VI level lymph nodes. The histological evaluation of these 74 FN cases based on initial FNAC interpretation resulted in 49 (66.2%) histologically proven malignant lesions, mostly represented by PTC and its subtypes, as well as infiltrative and minimally invasive encapsulated follicular variant of PTC. Thus, it appears that > 50% of the subcentimeter FN cases in this Italian series had a malignant histological diagnosis, including cases that even exhibited more aggressive features such as tall cell, solid, and hobnail components. Furthermore, 30.6% (15/49) of these malignant lesions had lymph node involvement, confirming that thyroid nodule size alone is not a significant parameter.

The current study has some limitations. Firstly, this is a retrospective study from a single tertiary medical center. Although our approach to incidental subcentimeter thyroid nodules is like that of other Italian centers, it may not be broadly adopted in other institutions around the world. Secondly, the use of different thyroid US classification systems is linked to different conclusions and hence different decisions about whether to perform FNAC. Thirdly, the diagnosis of subcentimeter thyroid lesions with cytomorphological features in favor of a FN is controversial in the field of thyroid cytology. Per the Italian cytology classification system, any evidence of nuclear atypia in the aspirate places the lesion in the TIR3B or FN category, which needs to be followed by surgery, while according to the Bethesda Thyroid System, nuclear atypia can also be detected in the AUS category, which portends a higher risk of malignancy [11, 12, 14].

As has been shown in several papers, the performance of ancillary techniques on cytological material has helped diagnostically resolve indeterminate cases [36–44]. In this series, most malignant cases confirmed by ancillary studies on FNAC residual material among the indeterminate lesions had a histological diagnosis of invasive and/or encapsulated follicular variant of PTC, for which *BRAF v600E* is frequently wild type. RAS mutations are often seen in both benign and malignant thyroid lesions and thus by themselves do not specify malignancy [36–44]. According to some previous studies, including those from our group, there is a correlation, up to 77% of the cases, between the morphology of FN resulting as malignant on histology and the concordant positivity of HBME-1 and Galectin-3 [24–28].

In detail, the evaluation of an immunopanel made up of HBME-1, Galectin-3, and VE1-BRAF in our series was able to highlight those eight cases that had a positive immunopanel for HBME-1 and Galectin-3, which is more likely associated with a malignant lesion [24–28]. These eight FN cases resulted in a histological follow-up diagnosis of PTC, five of which were classic PTC and two PTC with a tall cell component. Concerning the evaluation of VE1-BRAF, it was seen as moderate expression in only 10% of the neoplastic cells in three cases. According to the focal expression, but due to the high specificity of this genetic alteration for PTC and its subtypes, we decided to report it in the sample report, maintaining the diagnoses of FN for the limited expression. However, these three cases underwent a more personalized management defined by a total thyroidectomy plus VI level nodal dissection. Although ICC is unable to help make a definitive diagnosis, it serves as a useful pathology ancillary tool in the algorithmic work-up of subcentimeter thyroid lesions.

In conclusion, in our institution, the subcentimeter size of a thyroid nodule does not represent a limitation to perform FNAC. Rather, if a subcentimeter lesion has US criteria that are suspicious of malignancy, then FNAC is preferentially performed to further guide follow-up and, if necessary, surgical management.
